# Working hours of full-time hospital physicians in Japan: a cross-sectional nationwide survey

**DOI:** 10.1186/s12889-023-17531-5

**Published:** 2024-01-12

**Authors:** Soichi Koike, Hiroo Wada, Sachiko Ohde, Hiroo Ide, Kenichiro Taneda, Takeshi Tanigawa

**Affiliations:** 1 Division of Health Policy and Management, Center for Community Medicine, Jichi Medical University, 3311-1 Yakushiji, Shimotsuke, Tochigi, 329-0498 Japan; 2 Department of Public Health, Juntendo University School of Medicine, 2-1-1 Hongo, Bunkyo-Ku, Tokyo, 113-8421 Japan; 3 Graduate School of Public Health, St. Luke’s International University, 3-6-2 Tsukiji, Chuo-Ku, Tokyo, 104-0045 Japan; 4 Institute for Future Initiatives, The University of Tokyo, 7-3-1 Hongo, Bunkyo-Ku, Tokyo, 113-0033 Japan; 5 Department of Health and Welfare Services, National Institute of Public Health, 2-3-6 Minami, Wako, Saitama 351-0197 Japan

**Keywords:** Working hours, Physician work reform, Community healthcare, University hospitals

## Abstract

**Background:**

The culture of excessively long overtime work in Japan has not been recently addressed. New legislation on working hours, including a limitation on maximum overtime work for physicians, will be enforced in 2024. This study was performed to elucidate the working conditions of full-time hospital physicians and discuss various policy implications.

**Methods:**

A facility survey and a physician survey regarding physicians’ working conditions were conducted in July 2022. The facility survey was sent to all hospitals in Japan, and the physician survey was sent to all physicians working at half of the hospitals. The physicians were asked to report their working hours from 11 to 17 July 2022. In addition to descriptive statistics, a multivariate logistic regression analysis on the factors that lead to long working hours was conducted.

**Results:**

In total, 11,466 full-time hospital physicians were included in the analysis. Full-time hospital physicians worked 50.1 h per week. They spent 45.6 h (90.9%) at the main hospital and 4.6 h (9.1%) performing side work. They spent 43.8 h (87.5%) on clinical work and 6.3 h (12.5%) on activities outside clinical work, such as research, teaching, and other activities. Neurosurgeons worked the longest hours, followed by surgeons and emergency medicine physicians. In total, 20.4% of physicians were estimated to exceed the annual overtime limit of 960 h, and 3.9% were estimated to exceed the limit of 1860 h. A total of 13.3% and 2.0% exceeded this level only at their primary hospital, after excluding hours performing side work. Logistic regression analysis showed that male, younger age, working at a university hospital, working in clinical areas of practice with long working hours, and undergoing specialty training were associated with long working hours after controlling for other factors.

**Conclusions:**

With the approaching application of overtime regulations to physicians, a certain reduction in working hours has been observed. However, many physicians still work longer hours than the designated upper limit of overtime. Work reform must be further promoted by streamlining work and task-shifting while securing the functions of university hospitals such as research, education, and supporting healthcare in communities.

## Background

Regulation of working hours is critical not only from the viewpoint of workers but also from the viewpoints of employers and governments [1]. From the viewpoint of employers, the International Labour Office in Geneva published a research synthesis paper summarizing the direct effect of reduced working hours on improved worker productivity. Additionally, the paper discussed how longer working hours were associated with lower productivity as well as the indirect effects of working time on productivity and firm performance via overwork [2]. Issues relating to working time also affect society at large. Target 8.8 of the Sustainable Development Goals, adopted by the United Nations in 2015, has set a goal to protect labor rights and promote safe and secure working environments for all workers [3]. The government can use the working-time regulation policy to resolve social problems such as work–life balance, protection of health, and safety and well-being of workers [1].

The effects of excessive working hours on health have been thoroughly investigated in multiple studies. Systematic reviews and meta-analyses have shown that long working hours are associated with ischemic heart disease [4, 5], stroke, [3, 6, 7] and diabetes [8]. Long working hours are also known to be associated with depression, burnout, lower quality of life, and lower career satisfaction [9, 10]. One study showed that after continuously being awake for 17 h, participants’ cognitive psychomotor performance decreased to a level equivalent to the performance impairment observed at a blood alcohol concentration of 0.05%, the level at which alcohol intoxication is legally defined in many areas [11].

Long working hours among physicians are a major issue in Japan. A report on psychological stress reactions among non-physician workers indicated an increase in depressive symptoms [12] and death and suicide attempts among physicians [13]. Additionally, surveys on physicians’ long working hours [14] and experience of burnout [15, 16] have depicted an occupational threat to Japanese physicians [17].

The quality of care provided by physicians may be lowered by impaired cognitive performance due to continuous working hours, and this situation would eventually lead to negative effects on patients [18]. Governments of various countries are attempting to avoid this issue by enforcing regulations such as working hour restrictions for young physicians by limiting night shifts, limiting monthly night shifts, enforcing short consecutive night shifts, and limiting weekly working hours [19, 20].

Despite evidence that working hour regulations have a positive impact, several potential negative impacts of such regulations have also been noted, especially for young physicians. These impacts include discontinuity of care, an increased clinical workload of attending physicians, and decreased educational opportunities [21]. In addition, studies performed to date contain some design limitations, resulting in mixed effects on surgical experience and the quality of education [22]. Therefore, the results remain inconclusive.

The Labor Standards Law in Japan was amended in 2019, and limited overtime work with penalties was enforced. However, the enforcement of this regulation for physicians was postponed for 5 years under consideration of the Medical Practitioners' Act, which states that “a physician engaged in medical treatment shall not refuse medical treatment without just cause when requested to do so,” as well as the fact that local medical services were based on the premise of long working hours by physicians. During the grace period, the Government Panel on Physicians Work Reform was established. The panel stated the following: “It is necessary to share the current awareness that the medical care in our country is in a critical situation, supported by the self-sacrificing long working hours of physicians. Physicians work long hours compared with other occupations, especially young physicians in their 20 s and 30 s” [23]. The panel also discussed the details of the overtime cap and measures to ensure health. New overtime regulations for physicians will be introduced in the fiscal year 2024. For physicians who engage in clinical activities, an annual overtime limit of 960 h (Level A) will be applied. In addition, a maximum of 1,860 h per year will be allowed if annual excess working hours unavoidably exceed 960 h per year; this will be implemented to ensure the stability of the local healthcare delivery system (Level B) or to allow workers to intensively acquire and improve their skills (Level C) with additional health security measures [24–26].

Two nationwide surveys on physicians’ working status in Japan were conducted in 2016 [27] and 2019 [28]. According to the more recent survey in 2019, the average weekly working hours of full-time hospital physicians was 56.4 h. Among these physicians, 37.8% exceeded 60 h per week. The longest working hours were associated with surgery (61.9 h), followed by neurosurgery (61.9 h) and emergency medicine (61.0 h). Clinical residents worked 57.4 h per week. Physicians at a university hospital worked 49.2 h per week at their primary workplace (clinical activities, 35.6 h; research, 6.6 h; and education, 2.6 h).

Because the most recent survey was conducted before the details of the post-2024 system were decided, the level of interest among healthcare professionals and the working conditions of physicians are likely to have been different at that time. Updating the working hour data will provide a better understanding of the situation immediately before introduction of the new regulation and will help identify the remaining challenges ahead. The present study was performed to elucidate the working conditions of full-time hospital physicians and discuss various policy implications.

## Methods

**Fig. 1:**
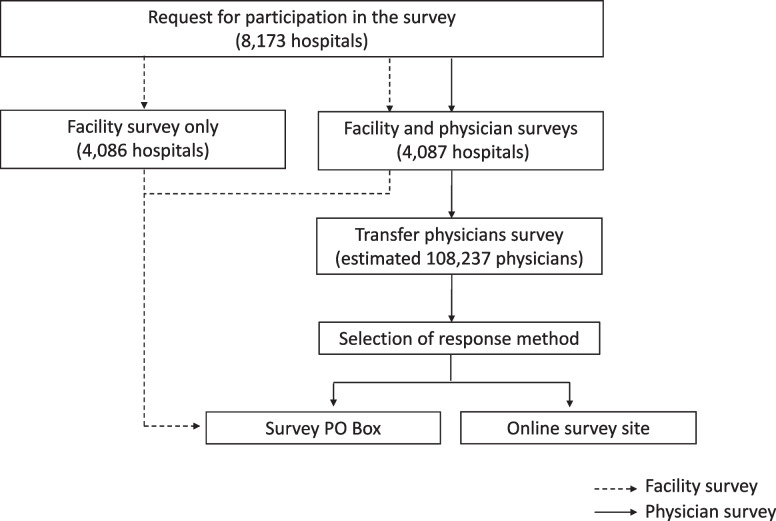
Study Design of Facility Survey and Physician Survey of Physicians’ Working Condition (Hospital). Two surveys were conducted: a facility survey to be answered by the facility staff and a physician survey to be answered by the physicians. For the facility survey, hospitals received a request for cooperation from the research team and were asked to respond by mail. For the physician survey, physicians received a request for cooperation through their hospitals and were asked to respond directly by mail or via a dedicated survey website of their choice. The facility survey was sent to all hospitals in Japan (*n* = 8,173). The physician survey was sent to all physicians (estimated *n* = 108,237) working in 50% of randomly selected hospitals (*n* = 4,087).

Two surveys on the working conditions of physicians were conducted in July 2022: a facility survey and a physician survey. The facility survey was sent to all hospitals in Japan (*n* = 8,173 hospitals), and the physician survey was sent through these facilities to all physicians working for half of the hospitals (randomly selected) (estimated *n* = 108,237 physicians). Both surveys were also conducted in clinics, geriatric healthcare facilities, and long-term care medical facilities (10% of randomly selected facilities and all physicians working for those surveyed facilities). The facilities that agreed to participate mailed the survey questionnaire back to the survey office. Physicians who agreed to participate responded directly to the survey office by mail or through a website dedicated to answering the survey (Fig. 1).

The facility survey included questionnaire items on attributes of the facilities as well as the status of work style reform. The physician survey included questionnaire items on the physicians’ attributes, status of side work, views on reforming the way they work, and working hours during a designated week (11–17 July 2022) by primary and secondary workplaces, by clinical activities and non-clinical activities (research, education, and other activities), and by holiday shift and night shift. These working hours were reported in 30-min increments.

Responses with missing data on sex, age, registration year as a physician, area of practice, and work type; responses sent through an incorrect path; and responses with inconsistent work hours by work type were excluded from the final analysis. Full-time hospital physicians were evaluated in all analyses.

Working hours were defined as the sum of clinical work hours, non-clinical work hours (excluding voluntary activities without a supervisor’s instruction), and standby hours on night shifts and holiday shifts (on-call standby hours outside the hospital were excluded from working hours). Hours that overlapped with clinical and non-clinical activities were considered as clinical activities.

Annual overtime hours for physicians who worked > 60 h per week were considered as annual excess work of 960 h, and annual overtime hours for physicians who worked > 78 h 45 min per week were considered as annual excess work of 1,860 h.

We presented descriptive statistics on working hours by physician attributes and performed a logistic regression analysis to assess factors associated with excess annual estimated overtime of > 960 and > 1,860 h. The dependent variables were whether annual overtime was > 960 or > 1,860 h. The independent variables were sex (male, female, or no response), age group (20 s, 30 s, 40 s, 50 s, or ≥ 60 s), main clinical area of practice (the respondents were asked to select the closest of 20 areas of practice listed in the questionnaire; if they considered that none of the areas corresponded to their area of practice, they were to select “other”), type of hospital (university hospital or non-university hospital), and specialty training status (in specialty training program or other). Comparisons of categorical and continuous variables between the two groups (university and non-university hospitals) were conducted using the chi-square test and Mann–Whitney U test, respectively. Statistical analysis was performed using IBM SPSS Statistics for Windows, Version 27.0. (IBM Corp., Armonk, NY, USA). A *P* value of < 0.05 was considered statistically significant.

## Results

A total of 16,214 hospital physicians responded to the questionnaires; thus, the estimated response rate was 15.0%. Among the respondents, 11,466 full-time physicians working at hospitals were used for the analysis. Physicians working for university hospitals comprised more female physicians, younger physicians, and physicians in specialty training than those working for other types of hospitals (Table 1).

**Table 1:** Characteristics of study participants

	Hospital total (*n* = 11,466)	University hospital (*n* = 2,608)	Non-university hospital (*n* = 8,858)	*P* value
**Sex**				
Male	8,983 (78.3)	2,015 (77.3)	6,968 (78.7)	0.02*
Female	2,434 (21.2)	588 (22.5)	1,846 (20.8)	
Other	49 (0.4)	5 (0.2)	44 (0.5)	
**Age, years**	47.3 ± 12.8	42.8 ± 10.0	48.7 ± 13.2	< 0.001***
**Area of practice**				
Internal medicine	3,637 (31.7)	789 (30.3)	2,848 (32.2)	< 0.001***
Surgery	1,403 (12.2)	311 (11.9)	1,092 (12.3)	
Pediatrics	673 (5.9)	156 (6.0)	517 (5.8)	
Obstetrics and gynecology	404 (3.5)	76 (2.9)	328 (3.7)	
Psychiatry	548 (4.8)	105 (4.0)	443 (5.0)	
Dermatology	217 (1.9)	88 (3.4)	129 (1.5)	
Ophthalmology	267 (2.3)	101 (3.9)	166 (1.9)	
Otolaryngology	252 (2.2)	106 (4.1)	146 (1.6)	
Urology	341 (3.0)	78 (3.0)	263 (3.0)	
Orthopedics	789 (6.9)	133 (5.1)	656 (7.4)	
Neurosurgery	445 (3.9)	67 (2.6)	378 (4.3)	
Plastic surgery	132 (1.2)	55 (2.1)	77 (0.9)	
Emergency medicine	217 (1.9)	60 (2.3)	157 (1.8)	
Anesthesiology	616 (5.4)	168 (6.4)	448 (5.1)	
Radiology	341 (3.0)	88 (3.4)	253 (2.9)	
Rehabilitation	228 (2.0)	18 (0.7)	210 (2.4)	
Pathology	167 (1.5)	67 (2.6)	100 (1.1)	
Laboratory medicine	32 (0.3)	6 (0.2)	26 (0.3)	
General practice	154 (1.3)	34 (1.3)	120 (1.4)	
Resident	346 (3.0)	39 (1.5)	307 (3.5)	
Others	257 (2.2)	63 (2.4)	194 (2.2)	
**Status of specialist training**				
Specialist trainee	1,345 (11.7)	388 (14.9)	957 (10.8)	< 0.001***

Data are presented as n (%) or mean ± standard deviation

^*^P < 0.05, ^**^P < 0.01, ^***^P < 0.001

Full-time hospital physicians worked 50.1 h per week. They spent 45.6 h (90.9%) at the main hospital and 4.6 h (9.1%) performing side work. Additionally, they spent 43.8 h (87.5%) on clinical work and 6.3 h (12.5%) on activities outside clinical work, such as research, teaching, and other activities (Table 2).

**Table 2:** Breakdown of working hours per week

	Hospital total (*n* = 11,466)	University hospital (*n* = 2,608)	Non-university hospital (*n* = 8,858)	*P* value
**Total working hours**	50.1 (100.0)	54.2 (100.0)	48.9 (100.0)	< 0.001***
Clinical activities	43.8 (87.5)	42.7 (78.8)	44.2 (90.3)	< 0.001***
Regular work shift	41.2 (82.2)	40.3 (74.4)	41.5 (84.8)	0.001**
Night and holiday shifts	2.6 (5.2)	2.4 (4.4)	2.7 (5.5)	< 0.001***
Non-clinical activities	6.3 (12.5)	11.5 (21.2)	4.7 (9.7)	< 0.001***
Research	1.7 (3.4)	4.4 (8.1)	0.9 (1.8)	< 0.001***
Education	0.7 (1.4)	1.7 (3.0)	0.4 (0.8)	< 0.001***
Training	2.1 (4.2)	3.0 (5.5)	1.9 (3.8)	< 0.001***
Others	2.3 (4.5)	3.2 (5.9)	2.0 (4.1)	< 0.001***
**Working hours, main workplace**	45.6 (90.9)	43.8 (80.8)	46.1 (94.2)	< 0.001***
Clinical activities	40.0 (79.8)	33.7 (62.1)	41.9 (85.6)	< 0.001***
Regular work shift	38.0 (75.7)	32.3 (59.6)	39.6 (81.0)	< 0.001***
Night and holiday shifts	2.0 (4.1)	1.4 (2.5)	2.2 (4.6)	< 0.001***
Non-clinical activities	5.5 (11.1)	10.2 (18.7)	4.2 (8.6)	< 0.001***
Research	1.5 (3.0)	4.0 (7.4)	0.8 (1.6)	< 0.001***
Education	0.6 (1.2)	1.5 (2.7)	0.4 (0.7)	< 0.001***
Training	1.8 (3.7)	2.5 (4.7)	1.6 (3.4)	< 0.001***
Others	2.0 (4.0)	2.8 (5.1)	1.8 (3.7)	< 0.001***
**Working hours, side work**	4.6 (9.1)	10.4 (19.2)	2.9 (5.8)	< 0.001***
Clinical activities	3.8 (7.7)	9.0 (16.7)	2.3 (4.7)	< 0.001***
Regular work shift	3.3 (6.5)	8.0 (14.8)	1.9 (3.8)	< 0.001***
Night and holiday shifts	0.6 (1.2)	1.0 (1.9)	0.5 (0.9)	< 0.001***
Non-clinical activities	0.7 (1.5)	1.4 (2.5)	0.5 (1.1)	< 0.001***
Research	0.2 (0.4)	0.4 (0.7)	0.1 (0.2)	< 0.001***
Education	0.1 (0.2)	0.2 (0.3)	0.1 (0.1)	< 0.001***
Training	0.3 (0.6)	0.5 (0.8)	0.2 (0.5)	< 0.001***
Others	0.2 (0.5)	0.4 (0.7)	0.2 (0.4)	< 0.001***

Data are presented as n (%)

^*^P < 0.05, ^**^P < 0.01, ^***^P < 0.001

**Fig. 2:**
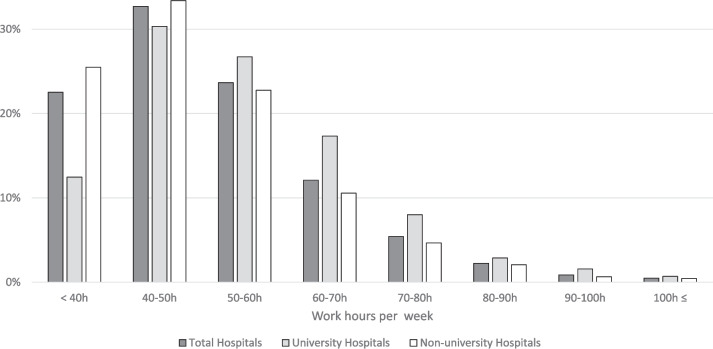
Distribution of Working Hours of Hospital Physicians by Hospital Type

The frequency distribution showed that 40 to 50 h of work per week was the most common. The work hours for 20.4% (2,344/11,466) of the physicians exceeded the estimated annual overtime work of 960 h, and the work hours for 3.9% (446/11,466) exceeded 1,860 h. If the work hours were limited to their principal workplace, 13.3% (1,526/11,466) and 2.0% (234/11,466) of the physicians exceeded the estimated annual overtime work of 960 and 1860 h, respectively (Fig. 2, Table 3).

**Table 3:** Estimated annual excess work hours and side work

	Principal workplace			*P* value
	Hospital total (*n* = 11,466)	University hospital (*n* = 2,608)	Non-university hospital (*n* = 8,858)	
**Annual excess work hours including side work**				
≤ 960 h	9,122 (79.6)	1,834 (70.3)	7,288 (82.3)	< 0.001***
> 960 to 1,860 h	1,898 (16.6)	627 (24.0)	1,271 (14.3)	
> 1,860 h	446 (3.9)	147 (5.6)	299 (3.4)	
**Annual excess work hours excluding side work**				
≤ 960 h	9,940 (86.7)	2,306 (88.4)	7,634 (86.2)	0.007**
> 960 to 1,860 h	1,292 (11.3)	262 (10.0)	1,030 (11.6)	
> 1,860 h	234 (2.0)	40 (1.5)	194 (2.2)	

Data are presented as n (%)

^*^P < 0.05, ^**^P < 0.01, ^***^P < 0.001

Neurosurgeons worked the longest hours, followed by general surgeons and emergency medicine physicians. Clinical residents worked 46.4 h per week, and those in specialty training programs worked 54.4 h per week (Table 4).

**Table 4:** Estimated work hours per week by area of practice

	Principal workplace			*P* value
	Hospital total (*n* = 11,466)	University hospital (*n* = 2,608)	Non-university hospital (*n* = 8,858)	
Internal medicine	49.6 ± 13.6	52.7 ± 13.0	48.7 ± 13.6	< 0.001***
Surgery	54.5 ± 15.2	60.4 ± 15.8	52.9 ± 14.7	< 0.001***
Pediatrics	50.3 ± 15.0	54.0 ± 14.6	49.2 ± 15.0	< 0.001***
Obstetrics and gynecology	52.3 ± 16.5	57.3 ± 15.0	51.1 ± 16.6	< 0.001***
Psychiatry	45.7 ± 13.7	52.8 ± 15.1	44.1 ± 12.8	< 0.001***
Dermatology	46.3 ± 12.8	49.9 ± 12.8	43.8 ± 12.2	< 0.001***
Ophthalmology	45.1 ± 11.9	49.6 ± 13.0	42.3 ± 10.2	< 0.001***
Otolaryngology	49.8 ± 13.2	54.8 ± 12.7	46.1 ± 12.2	< 0.001***
Urology	52.7 ± 12.6	59.6 ± 13.2	50.6 ± 11.7	< 0.001***
Orthopedics	51.6 ± 13.7	57.3 ± 13.7	50.4 ± 13.5	< 0.001***
Neurosurgery	56.4 ± 16.1	63.9 ± 14.9	55.1 ± 15.9	< 0.001***
Plastic surgery	50.0 ± 15.0	54.3 ± 18.3	46.8 ± 11.1	0.01*
Emergency medicine	54.1 ± 14.3	55.1 ± 13.2	53.7 ± 14.7	0.33
Anesthesiology	48.4 ± 12.6	52.6 ± 13.0	46.8 ± 12.1	< 0.001***
Radiology	46.5 ± 10.5	49.2 ± 12.2	45.5 ± 9.7	0.01*
Rehabilitation	44.6 ± 12.5	57.9 ± 20.5	43.4 ± 10.8	< 0.001***
Pathology	46.3 ± 12.7	51.3 ± 13.4	43.0 ± 10.9	< 0.001***
Laboratory medicine	38.9 ± 13.5	44.7 ± 17.3	37.5 ± 12.0	0.49
General practice	50.7 ± 14.2	51.2 ± 14.8	50.6 ± 14.0	0.91
Resident	47.9 ± 11.4	46.4 ± 11.7	48.1 ± 11.4	0.36
Others	46.4 ± 12.4	50.1 ± 12.2	45.2 ± 12.2	0.003**
Specialist trainee (relisted)	54.4 ± 14.8	55.3 ± 16.0	54.0 ± 14.3	0.52

Data are presented as mean ± standard deviation

^*^P < 0.05, ^**^P < 0.01, ^***^P < 0.001

The multiple logistic regression analyses showed that after adjustment for covariates, male, younger age (< 30 years), neurosurgery, obstetrics and gynecology, surgery, emergency medicine, urology, general practice, orthopedics, working at a university hospital, and being in a specialty training program were factors associated with annual overtime hours exceeding 960 h. Male sex, younger age (< 40 years), neurosurgery, obstetrics and gynecology, surgery, pediatrics, and working at a university hospital were associated with annual overtime hours exceeding 1,860 h (Table 5).

**Table 5:** Logistic regression analysis

	Estimated annual overtime hours > 960			Estimated annual overtime hours > 1,860		
	n	Adjusted OR (95% CI)	*P* value	n	Adjusted OR (95% CI)	*P* value
**Sex**						
Male	1,946/8,983	Reference		389/8,983	Reference	
Female	393/2,434	0.62 (0.54–0.7)	< 0.001***	55/2434	0.43 (0.32–0.58)	< 0.001***
Other	5/49	0.48 (0.19–1.22)	0.12	2/49	1.23 (0.29–5.17)	0.78
**Age, years**						
< 30	254/990	Reference		52/990	Reference	
30–39	684/2,590	0.76 (0.63–0.92)	0.005**	149/2,590	0.72 (0.51–1.02)	0.06
40–49	693/2,874	0.67 (0.55–0.81)	< 0.001***	127/2,874	0.52 (0.36–0.74)	< 0.001***
50–59	515/2,760	0.49 (0.40–0.60)	< 0.001***	88/2,760	0.36 (0.25–0.53)	< 0.001***
≥ 60	198/2,252	0.20 (0.16–0.25)	< 0.001***	30/2,252	0.14 (0.09–0.23)	< 0.001***
**Workplace**						
Non-university hospital	1,570/8,858	Reference		299/8,858	Reference	
University hospital	774/2,608	1.81 (1.62–2.02)	< 0.001***	147/2,608	1.54 (1.24–1.91)	< 0.001***
**Area of practice**						
Internal medicine	2,947/3,637	Reference		119/3,637	Reference	
Surgery	986/1,403	1.82 (1.57–2.11)	< 0.001***	100/1,403	2.26 (1.71–2.98)	< 0.001***
Pediatrics	529/673	1.17 (0.95–1.44)	0.13	32/673	1.58 (1.05–2.36)	0.03*
Obstetrics and gynecology	291/404	1.95 (1.53–2.48)	< 0.001***	24/404	2.35 (1.48–3.72)	< 0.001***
Psychiatry	476/548	0.63 (0.48–0.82)	0.001**	14/548	0.77 (0.44–1.35)	0.36
Dermatology	190/217	0.51 (0.33–0.77)	0.001**	4/217	0.52 (0.19–1.44)	0.21
Ophthalmology	240/267	0.42 (0.28–0.63)	< 0.001***	3/267	0.32 (0.10–1.02)	0.05
Otolaryngology	203/252	0.81 (0.58–1.13)	0.22	4/252	0.38 (0.14–1.04)	0.06
Urology	254/341	1.37 (1.05–1.79)	0.02*	10/341	0.80 (0.42–1.55)	0.52
Orthopedics	609/789	1.25 (1.03–1.51)	0.02*	34/789	1.27 (0.86–1.89)	0.23
Neurosurgery	282/445	2.69 (2.16–3.35)	< 0.001***	44/445	3.34 (2.31–4.82)	< 0.001***
Plastic surgery	108/132	0.75 (0.47–1.19)	0.22	9/132	1.84 (0.90–3.75)	0.09
Emergency medicine	147/217	1.69 (1.25–2.29)	0.001**	11/217	1.23 (0.65–2.34)	0.52
Anesthesiology	526/616	0.68 (0.53–0.87)	0.002**	14/616	0.68 (0.39–1.20)	0.18
Radiology	309/341	0.43 (0.29–0.62)	< 0.001***	3/341	0.26 (0.08–0.83)	0.02
Rehabilitation	212/228	0.44 (0.26–0.74)	0.002**	5/228	0.99 (0.40–2.46)	0.98
Pathology	146/167	0.57 (0.35–0.91)	0.02*	2/167	0.36 (0.09–1.49)	0.16
Laboratory medicine	29/32	0.62 (0.18–2.09)	0.44	1/32	1.50 (0.20–11.30)	0.69
General practice	117/154	1.34 (0.91–1.98)	0.14	5/154	0.94 (0.38–2.35)	0.90
Resident	297/346	0.45 (0.32–0.64)	< 0.001***	3/346	0.15 (0.04–0.47)	0.001***
Others	224/257	0.76 (0.51–1.11)	0.15	5/257	0.77 (0.31–1.92)	0.58
**Specialist training**						
No	730/3,646	Reference		134/3,646	Reference	
Yes	1,614/7,820	1.18 (1.06–1.31)	0.003**	312/7,820	1.17 (0.94–1.46)	0.16

*CI* Confidence interval, *OR* Odds ratio

^*^P < 0.05, ^**^P < 0.01, ^***^P < 0.001

## Discussion

In this study, we have elucidated the working status of Japanese physicians and identified factors associated with long working hours. Although some progress was observed, several challenges remain.

Compared with the 2019 survey, [26] the current survey shows a certain degree of reduction in working hours. In particular, the working hours per week of clinical residents was 9.5 h less (57.4–47.9 h). However, the composition of specialties with long working hours remained unchanged, and the research and education hours at university hospitals for university hospital-employed physicians outpaced the decrease in clinical hours. Moreover, the working hours for 20.4% of physicians exceeded the estimated annual overtime work of 960 h, and those for 3.9% of physicians exceeded 1860 h.

Previous studies have revealed factors associated with long working hours. Specialists caring for acutely ill patients or patients requiring intensive monitoring [29] and emergency care responsibilities [30] were associated with long work hours. Working hours are longer in rural areas than in urban areas for family medicine/general practice [31–33]. Because working hours depend on the nature of the disease being treated and the surrounding medical facilities, our results are consistent with those of previous studies. The Japanese government is promoting a policy that calls for the functional differentiation and reorganization of healthcare facilities and the promotion of work style reforms among healthcare professionals in an integrated manner. This policy requires a society-wide effort, not just efforts by individual physicians or healthcare facilities, and further calls for the introduction of shift work and the promotion of task-shifting with other professions. Our findings suggest that further efforts to implement the policy are necessary.

The role and function of university hospitals is another important issue to be discussed. University hospital physicians reportedly spend the majority of their time in patient care, whereas research and education are considered the roles of university hospitals [34]. In addition, the current study showed that the working hours of clinical residents decreased. Japanese residents spend significantly more time on patient care activities than on self-education and provide patient care while enduring sleep deprivation [35], suggesting that this downward trend in physician work hours may also be contributing to patient safety. Although reducing physicians’ overtime working hours is an important issue from the standpoint of ensuring physicians’ health, it is also important to evaluate the impact of this reduction on the training of young physicians, the research function at university hospitals, and the implementation of healthcare in communities supported by university hospitals. The present survey showed a decrease in research and education time at university hospitals, which could significantly impact the role that university hospitals should play in the future. Considering the decline in the number of research physicians in Japan [36], it is necessary to ensure that university hospitals do not simply become large operating hospitals because this would have a significant impact on the future of society.

The impact on community healthcare should also be noted. The results of this study show that university hospital physicians spend almost 20% of their working hours outside of their primary place of employment, including work hours that support community healthcare. Although efforts are being made to reduce working hours at universities, there is a possibility that physicians will try to meet the limitation of working hours by reducing their side work, as the calculation of the upper limit of working hours included side work. According to a national physician survey by the Ministry of Health, Labour and Welfare [37], 20.9% of Japanese physicians have side jobs. The two types of side work are voluntary side jobs and work that is dispatched by a university. The latter involves providing consistent medical care at smaller (usually regional) hospitals or performing surgery or other medical procedures [11]. Dispatching physicians is essential for maintaining community healthcare [12] and supplementing the income of young university hospital physicians who earn less than their counterparts in general hospitals [13]. This complexity adds challenges to resolving issues related to work hour regulations. Therefore, it is also necessary to examine how restrictions on physicians’ working hours impact local medical care.

### Limitations

This study had two main limitations. First, it involved a self-administered survey. With the approaching introduction of work reform, physicians are becoming more clearly aware of their working hours in anticipation of the introduction of an upper limit of overtime work. Although the government has already clarified the definition of working time [38], physicians who have not always strictly defined and managed their working hours in the past might not have reported their accurate working hours. In general, although self-administered surveys are easy to implement and require minimal capital investment, their accuracy has always been controversial. In recent years, methods for ascertaining work hours using electronic health records [39] and mobile apps [40, 41] have been introduced and are considered to be of some significance. Developing an easily implemented form of work hour ascertainment remains a challenge.

Second, the response rate was not markedly high. This may be due in part to the fact that the physician survey was conducted through medical institutions rather than directly asking physicians to participate. According to the Survey of Physicians, Dentists, and Pharmacists 2020, [32] the proportion of university hospital physicians among all hospital physicians in Japan was 26.2%, the average age was 45.1 years, the proportion of men was 76.2%, and the breakdown by area of practice was 34.2% for internal medicine, 11.2% for surgery, and 6.7% for orthopedics. Although the respondents of the current survey presented a lower percentage at university hospitals and a higher average age, the composition of respondents in this survey was considered to be a representative sample of the hospital physicians in Japan. However, in addition to these differences in participant demographics, unexpected bias between respondents and non-respondents may have affected the results.

## Conclusion

With the approaching application of overtime regulations to physicians, a certain reduction in working hours has been observed. However, many physicians still work longer hours than the designated upper limit of overtime work. It is necessary to further promote work reform through streamlining work and task-shifting while securing the functions of university hospitals such as research, education, and supporting healthcare in communities.

## Data Availability

The datasets generated and/or analyzed during the current study are available from the corresponding author on reasonable request.

## References

[1] International Labour Office. Report III (Part B). General Survey concerning working-time instruments - Ensuring decent working time for the future. Information and reports on the application of Conventions and Recommendations. International Labour Office, Geneva 2018. https://www.ilo.org/ilc/ILCSessions/previous-sessions/107/reports/reports-to-the-conference/WCMS_618485/lang--en/index.htm. Accessed 29 November 2023.

[2] Golden L. The effects of working time on productivity and firm performance: a research synthesis paper. International Labour Office. Conditions of work and employment series ; no 33, Geneva : ILO; 2012. https://labordoc.ilo.org/discovery/fulldisplay/alma994708473402676/41ILO_INST:41ILO_V2. Accessed 09 December 2023.

[3] United Nations. Department of Economic and Social Affairs. Sustainable Development Goals. https://sdgs.un.org/goals. Accessed 09 December 2023.

[4] Li J, Pega F, Ujita Y, Brisson C, Clays E, Descatha A, Ferrario MM, Godderis L, Iavicoli S, Landsbergis PA, Metzendorf MI, Morgan RL, Pachito DV, Pikhart H, Richter B, Roncaioli M, Rugulies R, Schnall PL, Sembajwe G, Trudel X, Tsutsumi A, Woodruff TJ, Siegrist J (2020). The effect of exposure to long working hours on ischaemic heart disease: A systematic review and meta-analysis from the WHO/ILO Joint Estimates of the Work-related Burden of Disease and Injury. Environ Int.

[5] Pega F, Náfrádi B, Momen NC, Ujita Y, Streicher KN, Prüss-Üstün AM, Descatha A, Driscoll T, Fischer FM, Godderis L, Kiiver HM, Li J, Magnusson Hanson LL, Rugulies R,  Sørensen K, Woodruff TJ, Technical Advisory Group (2021). Global, regional, and national burdens of ischemic heart disease and stroke attributable to exposure to long working hours for 194 countries, 2000–2016: A systematic analysis from the WHO/ILO Joint Estimates of the Work-related Burden of Disease and Injury. Environ Int.

[6] Descatha A, Sembajwe G, Pega F, Ujita Y, Baer M, Boccuni F, Di Tecco C, Duret C, Evanoff BA, Gagliardi D, Godderis L, Kang SK, Kim BJ, Li J, Magnusson Hanson LL, Marinaccio A, Ozguler A, Pachito D, Pell J, Pico F, Ronchetti M, Roquelaure Y, Rugulies R, Schouteden M, Siegrist J, Tsutsumi A, Iavicoli S (2020). The effect of exposure to long working hours on stroke: A systematic review and meta-analysis from the WHO/ILO Joint Estimates of the Work-related Burden of Disease and Injury. Environ Int.

[7] Virtanen M, Kivimäki M (2018). Long Working Hours and Risk of Cardiovascular Disease. Curr Cardiol Rep.

[8] Gilbert-Ouimet M, Ma H, Glazier R, Brisson C, Mustard C, Smith PM (2018). Adverse effect of long work hours on incident diabetes in 7065 Ontario workers followed for 12 years. BMJ Open Diabetes Res Care.

[9] Shanafelt TD, Balch CM, Bechamps GJ, Russell T, Dyrbye L, Satele D, Collicott P, Novotny PJ, Sloan J, Freischlag JA (2009). Burnout and career satisfaction among American surgeons. Ann Surg.

[10] Pulcrano M, Evans SR, Sosin M (2016). Quality of Life and Burnout Rates Across Surgical Specialties: A Systematic Review. JAMA Surg.

[11] Dawson D, Reid K (1997). Fatigue, alcohol and performance impairment. Nature.

[12] Ochiai Y, Takahashi M, Matsuo T, Sasaki T, Sato Y, Fukasawa K, Araki T, Otsuka Y (2023). Characteristics of long working hours and subsequent psychological and physical responses: JNIOSH cohort study. Occup Environ Med.

[13] Okawara M, Ishimaru T, Yoshikawa T, Kido M, Nakashima Y, Nakayasu A, Kimori K, Imamura S, Matsumoto K (2022). Working hours, side work, and depressive symptoms in physicians: A nationwide cross-sectional study in Japan. J Occup Health.

[14] Sekine M, Nishijima K, Nakagawa S, Suzuki Y, Murakami T, Kato Y, Umazume T, Tanaka H, Komatsu H, Doi K, Miura K, Kudo Y, Unno N, Kimura T, Enomoto T (2022). Challenges facing workstyle reform for Japanese obstetricians and gynecologists revealed from time studies. J Obstet Gynaecol Res.

[15] Morikawa M, Uechi T, Hanaki N, Goto Y, Funakoshi H, Takeuchi S, Mizobe M, Yajima T, Kondo Y, Tanaka H (2023). Burnout among Japanese emergency medicine physicians: A multicentric questionnaire study. Acute Med Surg.

[16] Nishimura K, Nakamura F, Takegami M, Fukuhara S, Nakagawara J, Ogasawara K, Ono J, Shiokawa Y, Miyachi S, Nagata I, Toyoda K, Matsuda S, Kataoka H, Miyamoto Y, Kitaoka K, Kada A, Iihara K, J-ASPECT Study Group (2014). Cross-sectional survey of workload and burnout among Japanese physicians working in stroke care: the nationwide survey of acute stroke care capacity for proper designation of comprehensive stroke center in Japan (J-ASPECT) study. Circ Cardiovasc Qual Outcomes.

[17] Hiyama T, Yoshihara M (2008). New occupational threats to Japanese physicians: karoshi (death due to overwork) and karojisatsu (suicide due to overwork). Occup Environ Med.

[18] Masterson MF, Shrichand P, Maniate JM (2014). Resident duty hours in Canada: a survey and national statement. BMC Med Educ.

[19] Maoz-Breuer R, Waitzberg R, Breuer A, Cram P, Bryndova L, Williams GA, Kasekamp K, Keskimaki I, Tynkkynen LK, van Ginneken V, Kovács E, Burke S, McGlacken-Byrne D, Norton C, Whiston B, Behmane D, Grike I, Batenburg R, Albreh T, Pribakovic R, Bernal-Delgado E, Estupiñan-Romero F, Angulo-Pueyo E, Rose AJ (2023). Work like a Doc: A comparison of regulations on residents’ working hours in 14 high-income countries. Health Policy.

[20] Temple J (2014). Resident duty hours around the globe: where are we now?. BMC Med Educ.

[21] Peets A, Ayas NT (2012). Restricting resident work hours: the good, the bad, and the ugly. Crit Care Med.

[22] Fletcher KE, Underwood W, Davis SQ, Mangrulkar RS, McMahon LF, Saint S (2005). Effects of work hour reduction on residents’ lives: a systematic review. JAMA.

[23] Ministry of Health, Labour and Welfare. Final Report of the Panel on Work Reform for Physicians 2019.03.29 https://www.mhlw.go.jp/stf/newpage_04273.html. Accessed 09 Dec, 2023. (in Japanese)

[24] Koike S (2021). The current state of work style reform of Japanese Physicians. Japanese J Health Econ Policy.

[25] Wada K, Endo M, Smith DR (2019). New Reforms to Limit the Excessive Working Hours of Japanese Physicians and Help Prevent Karoshi. J Occup Environ Med.

[26] Taneda K (2021). Labor reforms for physicians in Japan. J Natl Inst Public Health.

[27] Ministry of Health, Labour and Welfare. Survey of physicians' working conditions and working style. Study group for a vision of how doctors, nurses, etc. should work in light of new medical care. 15^th^ session conference document No.2. 2019.04.06. https://www.mhlw.go.jp/stf/shingi2/0000160696.html. Accessed 09 December 2023. (in Japanese)

[28] Tanigawa T. Outline of 2019 Survey of physicians' working conditions. Ministry of Health, Labour and Welfare. On the publication of the results of the “2019 Survey of physicians' working conditions” and “Survey on the Impact of Physician Workplace Reform on Community Health Care” 2020.7.31. https://www.mhlw.go.jp/stf/newpage_12705.html. Accessed 09 December 2023. (in Japanese)

[29] Leigh JP, Tancredi D, Jerant A, Kravitz RL (2011). Annual work hours across physician specialties. Arch Intern Med.

[30] Yu TH, Hou YH, Hsu HY, Chang RE (2022). Exploring Factors Associated With the Work Hours of Attending Physicians Working in Hospitals. Int J Health Policy Manag.

[31] Slade S, Busing N (2002). Weekly work hours and clinical activities of Canadian family physicians: results of the 1997/98 National Family Physician Survey of the College of Family Physicians of Canada. CMAJ.

[32] Weeks WB, Wallace AE (2008). Rural-urban differences in primary care physicians’ practice patterns, characteristics, and incomes. J Rural Health.

[33] Steinhaeuser J, Joos S, Szecsenyi J, Miksch A (2011). A comparison of the workload of rural and urban primary care physicians in Germany: analysis of a questionnaire survey. BMC Fam Pract.

[34] Nohara M, Yoshikawa T, Nakajima N, Okutsu K (2014). Hospital physicians perform five types of work duties in Japan: an observational study. BMC Health Serv Res.

[35] Deshpande GA, Soejima K, Ishida Y, Takahashi O, Jacobs JL, Heist BS, Obara H, Nishigori H, Fukui T (2012). A global template for reforming residency without work-hours restrictions: decrease caseloads, increase education. Findings of the Japan Resident Workload Study Group. Med Teach.

[36] Koike S, Ide H, Kodama T, Matsumoto S, Yasunaga H, Imamura T (2012). Physician-scientists in Japan: attrition, retention, and implications for the future. Acad Med.

[37] Ministry of Health, Labour and Welfare. Statistics of Physicians, Dentists and Pharmacists 2020. https://www.e-stat.go.jp/stat-search/files?page=1&toukei=00450026&tstat=000001135683. Accessed09 December 2023. (in Japanese)

[38] Director General, Labor Standards Bureau, Ministry of Health, Labour and Welfare. The concept of working hours and their self-improvement for physicians. Notification to Director General of Prefectural Labour Bureau. No. 0701–9. 2019.07.01 (in Japanese)

[39] Soleimani H, Adler-Milstein J, Cucina RJ, Murray SG (2021). Automating Measurement of Trainee Work Hours. J Hosp Med.

[40] Wang HH, Lin YH (2021). Assessing Physicians’ Recall Bias of Work Hours With a Mobile App: Interview and App-Recorded Data Comparison. J Med Internet Res.

[41] Jorgensen A, Savage NM, Sun X, Domson G (2022). Duty Hours Tracking - Is There an App for That?. J Med Educ Curric Dev.

